# Breadth-First Search-Based Single-Phase Algorithms for Bridge Detection in Wireless Sensor Networks

**DOI:** 10.3390/s130708786

**Published:** 2013-07-10

**Authors:** Vahid Khalilpour Akram, Orhan Dagdeviren

**Affiliations:** International Computer Institute, Ege University, Bornova, Izmir 35100, Turkey; E-Mail: orhandagdeviren@gmail.com

**Keywords:** wireless sensor networks, bridge detection, single-phase algorithm, network connectivity, distributed algorithm, breadth-first search

## Abstract

Wireless sensor networks (WSNs) are promising technologies for exploring harsh environments, such as oceans, wild forests, volcanic regions and outer space. Since sensor nodes may have limited transmission range, application packets may be transmitted by multi-hop communication. Thus, connectivity is a very important issue. A bridge is a critical edge whose removal breaks the connectivity of the network. Hence, it is crucial to detect bridges and take preventions. Since sensor nodes are battery-powered, services running on nodes should consume low energy. In this paper, we propose energy-efficient and distributed bridge detection algorithms for WSNs. Our algorithms run single phase and they are integrated with the Breadth-First Search (BFS) algorithm, which is a popular routing algorithm. Our first algorithm is an extended version of Milic's algorithm, which is designed to reduce the message length. Our second algorithm is novel and uses ancestral knowledge to detect bridges. We explain the operation of the algorithms, analyze their proof of correctness, message, time, space and computational complexities. To evaluate practical importance, we provide testbed experiments and extensive simulations. We show that our proposed algorithms provide less resource consumption, and the energy savings of our algorithms are up by 5.5-times.

## 1. Introduction

Wireless sensor networks (WSNs) are infrastructureless networks that consist of hundreds, even thousands, of sensor nodes that cooperate to perform sensing and communicating tasks. These nodes are low-cost, low-power multifunctional small devices. Healthcare, environmental control, military surveillance and target tracking are example application areas of WSNs [1–4]. Since sensor nodes are battery-powered and the transceiver is the dominant energy consumer, transmitted byte counts should be minimized to maximize the network lifetime.

Nowadays, WSNs are used in harsh environments, such as underground mines, oceans, tunnels and outer space [3,5,6]. Wireless communication in harsh environments has many challenges, such as channel (edge) failures, generally, as a consequence of the direct impact of the physical world. Some edges can have a critical role in data delivery operation. If one of these edges fails, the connectivity of the network is broken, and the network is divided into partitions. These type of edges are called bridges (cut edges), which should be detected, and preventions should be taken. Bridge detection is an important research area for various types of networks [7–13]. After bridge detection is completed, bridge neutralization algorithms can be applied [14].

**Figure 1.**
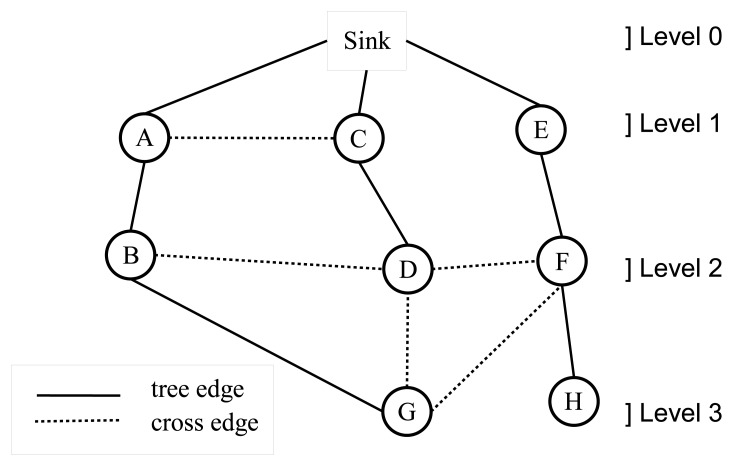
Breadth-first search (BFS) example.

A WSN can be modeled with a graph, *G* = (*V*,*E*), where *V* is the set of nodes and *E* is the set of edges. Breadth-first search (BFS) is a fundamental graph traversal algorithm, which starts from the sink node and search proceeds in a breadth-first manner [15]. Firstly, the sink node is visited; then, all neighbor nodes (*N_r_*) of the sink node are visited. In the following step, neighbor lists of nodes, ∈ *N_r_*, are visited sequentially. This operation continues, until there is no unvisited node in the graph. A sample BFS tree is given in Figure 1, where the order of the BFS traversal operation is: *sink*, *A*, *C*, *E*, *B*, *D*, *F*, *G* and *H*. After BFS is completed, the edges chosen by BFS are called tree edges, and the remaining edges are called cross edges. For example, the edges, (*A*,*B*), (*C*,*D*) and (*E*,*F*), are tree edges; the edges, (*A*,*C*), (*B*,*D*) and (*D*,*F*), are cross edges in Figure 1. BFS assigns a level value to each node, such as the level of the sink node is zero and the level of the other nodes are their shortest distance to the sink node, for example, *A*, *C* and *E* are nodes in level 1 and *G* and *H* are nodes in level 3 in Figure 1. This property is very important for routing in sensor networks, and there are many BFS-based approaches [16–20], since a shortest path tree rooted at the sink node can be constructed. BFS can be extended to detect bridges where routing and bridge detection can be accomplished at the same time [12,13]. In this study, we proposed bridge detection algorithms that are integrated with BFS and complete their operation within a single BFS execution.

The bridge detection algorithms proposed in the literature have some important disadvantages. Some of them should be implemented as a separate service [11,21]. Some of them have multi-phase execution [13,21]. Some of them cause transmission of long messages [12]. Regarding these deficiencies, we propose two energy-efficient bridge detection algorithms. The contributions of this paper are listed below:

- We propose an extended version of Milic's algorithm (E-MILIC). We propose two rules as the extension in order to reduce the message length of Milic's algorithm. We show from testbed experiments and extensive simulation results that E-MILIC consumes less resources than Milic's algorithm. Theoretical analysis and performance evaluations show that E-MILIC outperforms other bridge detection algorithms for graphs having a low cross edge count and a high diameter.
- We propose an ancestral knowledge-based bridge detection algorithm (ABIDE), which is a novel approach. ABIDE is integrated with the BFS, and it completes its operation within a single phase. Theoretical and practical studies show that for random graphs, ABIDE outperforms other bridge detection algorithms.

The rest of the paper is organized as follows. The network model and bridge detection problem are given in Section 2. The related work is summarized in Section 3. The proposed algorithms are described in Section 4. The theoretical analysis is given in Section 5. Testbed experiments and simulations are presented in Section 6. Finally, Section 7 provides conclusions.

## 2. Preliminaries

In this section, we explain the network model and the bridge detection problem.

### 2.1. Network Model

The following assumptions are made about the network, as in [11,13,22]:

- Each node has a distinct *node_id*.
- The nodes are stationary in the sensing area.
- Links between nodes are symmetric. Thus, if there is a link from *u* to *v*, there exists a reverse link from *v* to *u*.
- Nodes are not equipped with a position tracker, and they do not execute a localization algorithm; thus, each node does not know its position.
- All nodes have identical capabilities; they are equal in terms of processing capabilities, radio, battery and memory.
- Each node can send radio multicast messages to its immediate neighbors in its transmission range.
- Nodes are time-synchronized, where this can be achieved by implementing a protocol, such as proposed in [23].

**Figure 2.**
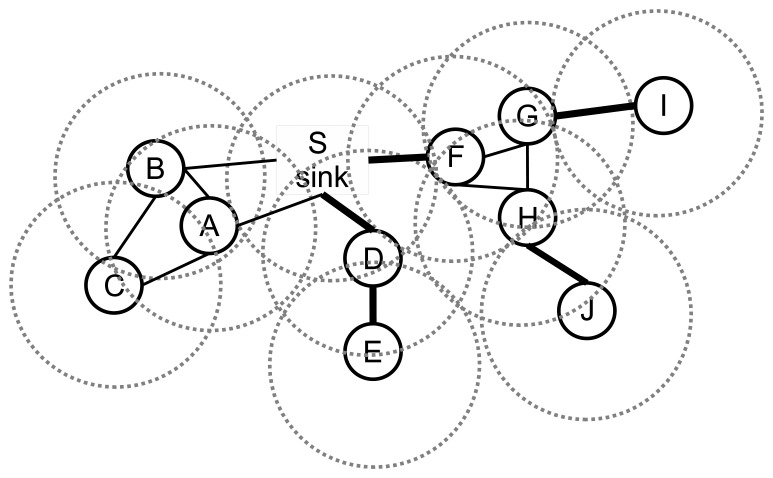
The graph model and the bridge problem.

The undirected graph model for the network is depicted in Figure 2, where there are 10 nodes, and the transmission ranges of the nodes are shown with dotted circles.

### 2.2. The Bridge Detection Problem

A bridge can be a link between a leaf node and its parent or it can be a link between a whole component to lower layers. An example sensor network is given in Figure 2. The bridges are shown with the bold lines. The edges, *DE*, *DS*, *FS*, *GI* and *HJ*, are bridges. If the edge, *DE*, fails, then leaf node, *E*, cannot transmit its packet to its parent. Similarly, node *I* and node *J* cannot relay their packets to their parents if the edges, *GI* and *HJ*, fail. If the edge, *DS*, fails, then both node *D* and node *E* cannot transmit their data to the sink node. If the edge, *FS*, fails, then node *F*, node *G*, node *H*, node *I* and node *J* cannot send their packets to the sink node, which leads to failure of 50% of the data packet transmissions. Therefore, bridges should be detected, and the preventions should be taken. In this paper, our focus is the detection of the bridges, and our objectives are listed below:

1. The bridge detection algorithm should be efficient in terms of message complexity and message size, since message transmission is the dominant factor of the energy consumption.
2. Routing is crucial for data delivery and data aggregation [24], and it is a fundamental service for sensor networks. If the bridge detection can be integrated with the routing operation, it can introduce a less total cost to the network. Preferably, the bridge detection approach should be a single-phase algorithm in order to consume less energy and time.
3. The bridge detection algorithm should be distributed, since the sink node may initiate the bridge detection algorithm at any time and locally.
4. In order to interface various medium access control (MAC) and physical layer standards [25], the bridge detection algorithm should be independent from the underlying protocols.
5. If location information is already available, a location-based cut detection algorithm can be used. However, for many sensor network setups, accurate localization cannot be afforded, due to the economic costs of GPS receivers, environmental effects (heat, wind, obstacles, *etc.*) on distance estimations and an insufficient number of anchor nodes. Since our concern is sensors for harsh-environment applications, we believe that if cut vertex detection service is independent from location information, then it can be integrated into many sensor network setups without the preconfiguration of locations.

## 3. Related Work

Depth-first search (DFS) is a fundamental technique that can be used for bridge detection [7,12]. Distributed DFS algorithms [26-32] can be modified to detect bridges by using the rules proposed by Tarjan [7]. Cidon [28], Hohberg [33], Chaudhuri [34], Tsin [32] and Turau [11] proposed distributed DFS-based algorithms for bridge and cut vertex (a vertex whose removal breaks the connectivity of a graph) detection algorithms. Turau proposed an algorithm [11] that is designed to detect bridges in sensor networks and that is an extended version of Cidon's [28] and Tsin's [32] algorithms. The algorithm transmits *4E* messages with a O(log_2_(*N*)) size, where *E* is the number of edges and *N* is the total node count, in the worst case. Although this algorithm completes its execution within a single DFS execution phase, if the medium access control (MAC) layer does not provide an edge-based sleep schedule, the upper bound for the received and overhead messages is as much as O(*δ*^2^*N*). Our algorithms have Θ(*N*) sent message complexity and O(*δN*) received and overheard message complexity. We show in this paper, with extensive simulations, that our algorithms are practically favorable. Besides, DFS cannot be used to construct a routing infrastructure, since then, it should be implemented as a separate service.

BFS is a very important algorithm for routing in various networks, and distributed versions of BFS are proposed [16-20]. A well-known greedy algorithm is applied for synchronous networks [18]. The message complexity of this algorithm is O(*N*), and the time complexity is O(*D*), where *D* is the diameter of the network. The BFS algorithms mentioned so far are not adequate for finding bridges. Although Liang's BFS-based algorithm detects bridges, it can run on permutation graphs, and it cannot be generalized [8]. Thurimella proposed a BFS-based bridge detection algorithm in which each node is assumed to know the whole topology [9], where it is not suitable for WSNs. The algorithms mentioned in this paragraph are not suitable for energy-efficient bridge detection in WSN, so they are out of our scope.

Pritchard proposed a three-phase distributed algorithm for bridge detection [21]. In the first phase, the nodes find a spanning tree in which BFS can be used. In the second phase, the algorithm computes preorder labels and subtree sizes. In the third phase, each node classifies its parent edge's states. Dagdeviren proposed an improved version of Pritchard's algorithm that completes within two phases [13]. Although these algorithms detect bridges in a distributed manner, they are multi-phase algorithms, and they are out of scope of this paper.

Dagdeviren proposed the ENBRIDGE algorithm for energy-efficient bridge detection [13]. The algorithm has two phases. In the first phase, nodes execute five rules on two-hop neighborhood information. The second phase is not executed, only if all edges are classified in the first phase. The second phase is an improved DFS-based algorithm. Similar to the other previously mentioned algorithms, this algorithm has multi-phase execution, and it is out of our scope. The proposed algorithms in this paper are energy- and time-efficient, and they complete within one phase.

Milic proposed a BFS-based bridge detection algorithm for wireless *ad hoc* networks (MILIC) [12]. The algorithm is fully integrated with the BFS and benefits from the radio multicast communication of the sensor network environment. The forward step of Milic's algorithm is nearly the same as the standard BFS. The nodes store a list of cross edges that they found or received in the backward step of the algorithm. The nodes append cross edges to the messages and send them to their parents. Although Milic's algorithm completes its operation within a single BFS execution phase, the message size can be large, since it depends on the number of cross edges. In this paper, we firstly propose an extended version of Milic's algorithm in order to reduce the message size by eliminating some of the cross edges. Our second proposed algorithm uses ancestral knowledge instead of a cross edge list. Besides, in this paper, we simulate Milic's algorithm against various network topologies and show that our proposed algorithms are favorable. The algorithms covered so far exactly find bridges without localization. In this study, we omit localization-based and probabilistic bridge detection and cut vertex detection algorithms, such as [14,35-39].

## 4. Proposed Algorithms

In this section, we will introduce our proposed E-MILIC and ABIDE algorithms.

### 4.1. Extended MILIC Algorithm

Although the MILIC algorithm completes its operation within a BFS execution session, the transmitted byte count can be high for energy-efficient sensor networks. In order to reduce transmitted byte count, we propose the extended-MILIC (E-MILIC) algorithm by adding two new rules to the MILIC algorithm as listed below:

- *Rule 1*: When the node, *n*, detects a cross edge, (*n*,*x*), it only transmits *x* instead of (*n*,*x*) to its parent, *p*. After node *p* receives this message from node *n*, it can simply decide that *x* represents the edge, (*n*,*x*), since the message is originated from node *n*. This rule reduces transmitted byte count in two situations. Firstly, since the MAC and physical layer include the source ID field, the routing layer packets of node *n* do not need to include the ID field for each (*n*,*x*) edge; thus, duplicate transmissions of ID fields are prevented. Secondly, if an edge-coloring-based MAC layer is used, then there is no need to transfer the ID field for all layers. The reason is that each node schedules its transmission times and can find the source of a packet by using its schedule.
- *Rule 2*: After the node, *n*, collects cross edges from its children, it runs a central *k*-cycle detection algorithm on the undirected graph, *G* = (*V*,*E*), where *E* is composed of incident cross edges, collected cross edges, the (*n*,*p*) edge and (*i*,*p_i_*) edges, where *i* is a neighbor of *n* and *p_i_* is its parent. The *V* is the set of incident vertices of the edges in *E*. These edges can be acquired by the overhearing method. The cycle detection algorithm detects *k*-cycles, where *k* ∈ {3,4} and the cycle should have exactly one cross edge.

**Figure 3.**
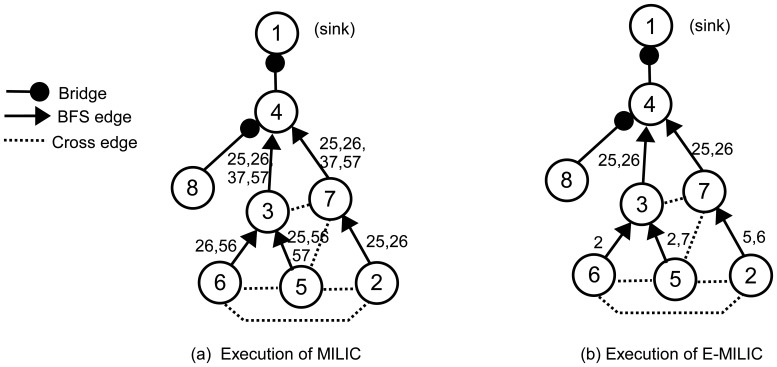
MILIC and extended-MILIC (E-MILIC) examples.

To explain these rules clearly, we give an example operation in Figure 3. In Figure 3a, an execution of the MILIC algorithm is given. We assume that each edge can can be represented with 2log_2_(*N*) bits. Firstly, node 2 sends its cross edges (the edge, (2,5), and the edge, (5,6)) to its parent, where the information of two edges are transmitted. Concurrently, node 5 and node 6 send their cross edges to their parent. These operations cause transmission of seven cross edges. When node 3 receives (2,6) and (3,6) from node 6 and (2,5), (5,6) and (5,7) from node 5, it matches the (5,6)'s from both lists and deletes them from the lists. Node 3 merges the two lists and adds (3,7) to this list. Node 3 transmits (2,5), (2,6), (3,7) and (5,7) to its parent, node 4. Concurrently, node 7 receives the message of node 2, appends (3,7) and (5,7) to node 2's list and sends it to node 4. Since node 8 has no cross edge, it sends an empty list, which indicates that (4,8) is a bridge. When node 4 receives both node 3 and node 7's messages, it matches (2,5), (2,6), (3,7) and (5,7) and deletes them from both lists. Node 4 sends an empty list to node 1, and (1,4) is classified as a bridge. Since 15 cross edge information is transmitted, the transmission overhead of the MILIC algorithm is 30log_2_(*N*) bits. Figure 3b shows the transmissions when we apply the proposed rules, where the transmission overhead is reduced to the 13log_2_(*N*) bits. Node 2, node 5 and node 6 use Rule 1 and send only one incident vertex of each edge. Node 5 and node 6 do not transmit the edge, (5,6), by finding a three-cycle, such as (5,6), (3,6) and (3,5), as given in Rule 2. Similarly, node 3 and node 7 do not transmit the edges, (3,7) and (5,7).

The message size of E-MILIC depends on the count of cross edges. In the following section, we will introduce a new algorithm, whose transmitted byte count is independent from cross edge count.

### 4.2. Ancestral Knowledge-Based Bridge Detection Algorithm

In E-MILIC algorithm, nodes process and send cross edge information to detect bridges. This may cause the transmission of large packets when the cross edge count is high. In order to reduce energy consumption for this case, we propose the ABIDE algorithm, which uses ancestral knowledge for bridge detection in WSNs. The algorithm is fully integrated to the BFS, where it completes its execution by the end of a single BFS phase execution. The main idea of the proposed algorithm is that the nodes process and send their ancestor's information to detect bridges. The algorithm has two steps: forward and backward steps. In the forward step, the spanning BFS tree, *τ*, is created by broadcasting *forward* messages between all nodes in a WSN. In the backward step, bridges are detected by convergecasting *backward* messages to the sink node, and the execution is terminated.

**Figure 4.**
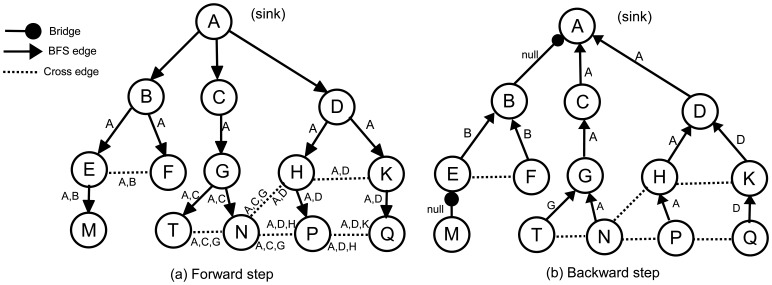
Transmitted messages in ancestral knowledge-based bridge detection algorithm (ABIDE) algorithm.

In the forward step of the algorithm, the sink node, *S*, broadcasts a *forward* message to its neighbors to construct *τ*. Initially, all nodes are *unvisited*. After receiving the first *forward* message, each node selects the sender as its parent, marks itself as *visited* and multicasts *forward* to its neighbors. We refer to the parent identifier of node *α* in the *τ* with *ρ*(*α*). Before multicasting, node *α* appends its parent identifier to the ancestor list, (Λ(*α*)), in the *forward* message; hence, a parent can find its ancestors by checking the ancestor list in the incoming *forward* messages. Λ(*α*) is the ancestor of node *α* in *τ*, which is ordered from *S* to *α*. In other words, Λ(*α*) shows the path of the *forward* message starting from *S* and ending at *α*. For example, in Figure 4a, Λ(*P*) is [*A*,*D*,*H*] and Λ(*N*) is [*A*,*C*,*G*]. Since the sink node does not have any ancestor, Λ(*S*) = ∅. Λ(*α*) is constructed by concatenating *ρ*(*α*) to the end of Λ(*ρ*(*α*)). Each *forward* message, *fm*, will carry a copy of its sender's ancestors, which is denoted by Λ(*fm*).

Obviously, the first common ancestor of all nodes is the sink, so that, in each Λ, the first element is always the sink. It is quite possible that the ancestors of two arbitrary nodes become identical, for example, in Figure 4a, Λ(*N*) = Λ(*T*) = [*A*,*C*,*G*]. The last common ancestor of *N* and *T* is *G*. We can write Λ(*P*)∩Λ(*Q*) = [*A*,*D*], so that the last common ancestor of *P* and *Q* is *D*. We define Λ(*α*)⋏Λ(*β*) as the last common ancestor in Λ(*α*)∩Λ(*β*). As we mentioned earlier, each sender puts its ancestor list into *forward* message. Therefore, if *α* receives a *forward* message, *fm*, from *β*, we can write: Λ(*α*)⋏Λ(*β*) = Λ(*α*)⋏Λ(*fm*).

Whenever a visited node, *α*, receives a *forward* message, *fm*, from node *β*, where *fm.parent* ≠ *α*, it can realize that the links to *β* and *ρ*(*α*) are not bridge and that *α* and *β* belong to a cycle. We define the identifier of a cycle as the starting element of the cycle. In Figure 4a, for example, the identifiers of cycle [*B*,*E*,*F*,*B*] (the underlying graph is undirected) is *B*, and the identifier of cycle [*A*,*D*,*H*,*P*,*N*,*G*,*C*,*A*] is *A*. Generally, if *α* and *β* belong to the same cycle, the identifier of this cycle is Λ(*α*)⋏Λ(*β*). *α* and *β* send Λ(*α*)⋏Λ(*β*) to their parents in the backward step to inform them about the cycle. A node may detect more than one cross edge and, therefore, may find more than one cycle. This may cause a parent to receive more than one cycle identifier from its children. For example, in Figure 4a, node *P* has two cross edges and, consequently, two cycles, which can be found by Λ(*P*)⋏Λ(*Q*) = *D* and Λ(*P*)⋏Λ(*N*) = *A*. This means that *P* creates two cycles, where the first one is closed by *D* and the second one is closed by *A*, so that {*A*,*D*} is the cycle identifier set in *P*. We formally define the set of cycle identifiers as in Definition 1.

#### Definition 1

Δ*(α) is a set of cycle identifiers that are formed by the* ⋏ *operation on* Λ*(α) and* Λ*(fm_r_), where fm_r_ is a received forward message, whose source is not ρ(α). This can be defined as* Δ*(α)*=∪*_r≠ρ(α)_* {Λ*(α)*⋏Λ*(fm_r_)*}.

In fact, if a node detects various cycles, it is sufficient to send the cycle identifier of the longest cycle to the parent, because it will cover other cycles. For example, sending *D* by *P* informs *H* and *D* that they belong to a cycle, but sending *A* informs *H*, *D* and *A* about the bigger cycle. The identifier of the biggest cycle in Δ(*α*) is the front element in Λ(*α*). We define *front*(Λ(*α*),Δ(*α*)) as the identifier of the front node of Λ(*α*) in Δ(*α*).

In the ABIDE algorithm, a leaf node in *τ* starts the backward step as follows: If Δ(*α*) is empty, then *α* marks *ρ*(*α*) as a bridge and sends *null* as *cycleID* to *ρ*(*α*). Otherwise, it marks *ρ*(*α*) as a non-bridge and sends *front*(Λ(*α*), Δ(*α*)) as *cycleID* to *ρ*(*α*). If *β*, which is not a leaf node, receives a *backward* message, *bm*, from *α*, then it executes the following rules:

- *Rule 1: If bm.cycleID* = *null, then the link toαis marked as a bridge*.
- *Rule 2: Else, if bm.cycleID* = *β, then the link toαis marked as a non-bridge*.
- *Rule 3: Else, the links to α and ρ (β) are marked as non-bridges. Furthermore, bm.cycleID is appended to* Δ*(β)*.

The pseudocode of the ABIDE algorithm is given in Algorithm 1. Figure 4 illustrates the exchanged messages in the ABIDE algorithm. Figure 4a shows the ancestor lists in the *forward* messages, and Figure 4b shows the cycle identifiers that are returned in the *backward* messages. The links between *AB* and *EM* are bridges. Except the first *forward* message, which is sent by *E*, the node, *M*, does not receive any additional *forward* messages. Therefore, Λ(*M*) = [*A*,*B*,*E*] and Δ(*M*) = ∅. Node *M* does not have any child and starts the backward step. It marks the link to *E* as a bridge and sends *backward(null)* to *E*, becausefront(Λ(*M*),Δ(*M*)) = ∅. Node *E* receives the first *forward* message from *B* and sets it as the parent. When node *E* receives the second *forward* message from *F*, it finds the cycle identifier as Λ(*E*)⋏Λ(*F*) = [*A*,*B*]⋏[*A*,*B*] = *B* and appends it to Δ(*E*). Upon receiving *null* from *M*, node *E* marks the link to *M* as a bridge, finds *front*(Λ(*E*),Δ(*E*)) = *front*([*A*,*B*], [*B*]) = *B* and sends *backward(B)* to *B*. In the same manner, node *F* sends *backward(B)* to *B*. Node *B* receives two *backward(B)* messages from its children. According to Rule 2, it marks both links as non-bridges, but does not append any value to Δ(*B*). Node *B* does not receive additional *forward* messages, and therefore, Δ(*B*) and *front*(Λ(*B*),Δ(*B*)) become empty. Hence, *B* marks the link to *A* as a bridge and sends *backward(null)* to *A*.

As previously mentioned, the node, *P*, in Figure 4 sets Λ(*P*) to [*A*,*D*,*H*]. It receives *forward([A*,*C*,*G])* from *N* and *forward([A*,*D*,*K])* from *Q*. [*A*,*D*,*H*]⋏[*A*,*C*,*G*] = *A* and [*A*,*D*,*H*]⋏[*A*,*D*,*K*] = *D*, so Δ(*N*) = [*A*,*D*] and *front*(Λ(*P*),Δ(*P*)) = *front*([*A*,*D*,*H*], [*A*,*D*]) = *A*. Therefore, node *P* marks its parent link as a non-bridge and sends *backward(A)* to *H*. According to Rule 3, the node, *H*, marks the links to *P* and *D* as non-bridges and sends *backward(A)* to *D*. The same process continues, until reaching the sink node. The following section introduces the theoretical analysis of the algorithms.

## 5. Analysis

In this section, we will analyze the proof of correctness, message, time, space and computational complexities of the ABIDE algorithm. At the end of this chapter, we will give the theoretical bounds of the E-MILIC algorithm.

### 5.1. Proof of Correctness

#### Assertion 1

*A node is apart of a cycle if it receives more than one forward message*.

##### Proof

We know that the algorithm is started by the sink node of the BFS tree. Additionally, we know that each node sends exactly one *forward* message when it receives a *forward* message from one of its neighbors. Therefore, there is a path between the sink and every node that broadcasts a *forward* message. Receiving a *forward* message from two distinct neighbors, *α* and *β*, in node *γ*, means that *γ* creates a path between *α* and *β*, which are previously connected to the sink node. Therefore, *α*, *β*, *γ* and the sink nodes create a cycle.

#### Assertion 2

*The beginning point of cycle c, which is detected by receiving a forward message fromβandα, is* Λ*(α)*⋏Λ*(β)*.

##### Proof

**Figure 5.**
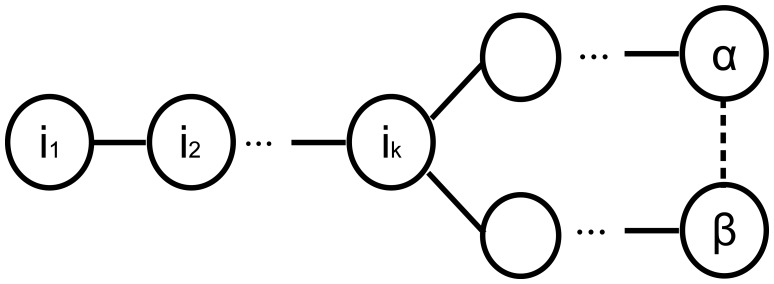
The case in which node *α* and node *β* have *k* common parents.

Let Λ_[*k*]_(*α*) be the *k^th^* node in the obtained BFS tree of node *α*. If *α* and *β* have *k* common parents in the BFS tree, as shown in Figure 5, then the first *k* elements in Λ(*α*) and Λ(*β*) are equal and Λ(*α*)⋏Λ(*β*) = Λ_[*k*]_(α) = Λ_[*k*]_(*β*), because Λ is ordered from the sink to the last parent of a node. According to Assertion 1, if α receives a non-first *forward* message from *β*, then we have a cycle that is started from ancestor *c* of both nodes. If we suppose that *c*≠ Λ(*α*)⋏Λ(*β*), then there must be another node, *m*, which Λ(*α*)⋏Λ(*β*) = *m*. In this case, the level of *m* must be greater than the level of *c*, which contradicts the ordered Λ hypothesis.


**Algorithm 1** ABIDE Algorithm.
 **message formats**: Forward(message type, sender, parent, list), Backward(message type, sender, destination, cycleID) **initially:** id is the unique node identifier; visited←false; cycleID←null; parent←null; backwardCount←childCount←0; cycleIDset←ancestors←∅ sink node multicasts Forward(forward type,sink,null,null) to neighbors **upon** a node receives a message  **if** msg.type = Forward **then call** ReceiveForward(msg)  **else if** msg.type = Backward **then call** ReceiveBackward(msg) **end if** **end upon** **procedure** ReceiveForward(Message msg)  **if** visited = false **then**   visited←true; parent←msg.sender; ancestors←msg.ancestors; ancestors.append(parent)   **multicast** Forward(forward type,id,parent,ancestors); **start** BackwardTimer  **else**   **if** msg.parent = id **then** childCount←childCount+1   **else** cycleIDset.add(ancestors⋏msg.ancestors); **mark** the link to msg.sender as non-bridge   **end if**  **end if** **end procedure** **upon** BackwardTimer expires  **if** childCount = 0 and id≠sink **then call** SendBackward() **end if** **end upon** **procedure** SendBackward()  **if** cycleIDset is empty **then**   **mark** the link to parent as bridge; **send** Backward(backward_type,id,parent,null) to parent  **else**   **mark** the link to parent as non-bridge; cycleID ←front(ancestors,cycleIDset)   **send** Backward(backward type,id,parent,cycleID) to parent  **end if** **end procedure** **procedure** ReceiveBackward(Message msg)  backwardCount←backwardCount+1  **if** msg.cycleID = null **then mark** the link to msg.sender as bridge  **else**   **mark** the link to msg.sender as non-bridge   **if** msg.cycleID≠id **then** cycleIDset.add(msg.cycleID) **end if**  **end if**  **if** backwardCount = childCount **then call** sendBackward() **end if** **end procedure**


Before presenting the next assertion, we define the edge set of a path between two node in a BFS tree in Definition 2.

#### Definition 2

*ζ_α_(x) is the set of edges that create a path from α to x in τ*.

In Figure 4, for example, *ζ_T_*(*C*) = [*TG*,*GC*] and *ζ_Q_*(*A*) = [*QK*,*KD*,*DA*]. Using the *ζ* definition, we can present the next assertion as follows.

#### Assertion 3

*For each i* ∈ Δ(*α*), *all edges in the ζ_α_(i) are non-bridges*.

##### Proof

According to Definition 1 and Assertion 2, there exists a cycle between *α* and each *i* ∈ Δ(*α*). This means that there are at least two paths between *α* and each *i* ∈ Δ(*α*), one of which is *ζ_α_*(*i*). Therefore, we can reach to each *i* ∈ Δ(*α*) by using more than one path, and therefore, all edges in the *ζ_α_*(*i*) are non-bridges.

#### Assertion 4

*Let x* = *front*(Λ*(α*),Δ*(α)),then* ∀*i* ∈ Δ(*α*):*ζ_α_*(*i*) ⊆ *ζ_α_(x)*.

##### Proof

All elements in Λ(*α*) are ordered from the sink to *α*. This means that all nodes in Λ(*α*) are ordered from the lowest level to the highest level in *τ*. Therefore, Λ_[*i*]_(*α*) is in a lower level than Λ_[*j*]_(*α*) in *τ*, if and only if *i* < *j*. Let *i_k_* be the *k^th^* element of Λ(*α*). This means that *i_k_* is one of the ancestors of *α*, which is located at level *k* in *τ*. Obviously, *ζ_α_*(*i_k_*) = ([*i_k_*, *i_k_*+ 1]) + *ζ_α_*(*i_k_* + 1), and therefore, *ζ_α_*(*i_k_* + 1)⊂*ζ_α_*(*i_k_*) is true, which can be generalized as ∀*i and* ∀*j*>*i*:*ζ_α_*(Λ_[*j*]_ (*α*))⊂*ζ_α_*(Λ_[*i*]_(*α*)). We know *front*(Λ(*α*),Δ(*α*)) has the least index in Λ(*α*) among other items in Δ(*α*). In other words, if we suppose *x* = *front*(Λ(*α*),Δ(*α*)), then ∀*i* ∈ Δ(*α*): index(*x*) ≤ index(*i*) in Λ(*α*). Therefore, we can write ∀*i* ∈ Δ(*α*) : *ζ_α_*(*i*) ⊆ *ζ_α_* (*x*), which implies assertion.

#### Assertion 5

*The link between α and ρ(α) is a bridge if and only if* Δ*(α)* = ∅.

##### Proof

The number of elements in Δ(*α*) may increase in two cases. In the first case, *α* receives a *forward* message from its neighbor, which is not its parent. In the second case, *α* receives a *backward* message with value, *x*, from its child, which *x* ≠ *α* and *x* ≠ *null*. In the first case, according to Assertions 1-3, the link between *α* and *ρ*(*α*) is not a bridge. In the second case, *x* is the identifier of a node that is in a lower level in *τ*. This means that *α* and *ρ*(*α*) are part of a cycle that is closed at *x*. Therefore, in this case, also, the link between *α* and *ρ*(*α*) is not a bridge. In reverse, suppose that Δ(*α*) = 0 and the link between *α* and *ρ*(*α*) is not bridge. Therefore, there must be a cycle that *α* and *ρ*(*α*) belong to. According to Definition 1, Δ(*α*) is a set of cycle identifiers, of which *α* and *ρ*(*α*) are parts of them. Therefore, Δ(*α*) = ∅ means that such a cycle does not exist in *τ*, which contradicts our assumption.

#### Theorem 1

*The ABIDE algorithm detects all bridge and non-bridge edges correctly*.

##### Proof

In the forward step of the ABIDE algorithm, each node divides its adjacent edges into three groups, which are: child link(s), parent link and cross link(s). A child link of node *α* connects node *α* to one of its children. The parent link connects node *α* to *ρ*(*α*). A cross link is an edge that *α* receives as a non-first *forward* message. In the backward step, each node decides whether these links are bridges or not. All cross links are non-bridges, because according to Assertion 1, cross links create a cycle in the BFS tree. According to Assertion 5, *α* can decide about *ρ*(*α*)'s link using Δ(*α*). According to Assertion 3, all links from *α* to each node in Δ(*α*) are non-bridges, and according to Assertion 4, sending *front*(Λ(*α*),Δ(*α*)) along the *backward* messages informs this matter to all nodes in Δ(*α*). If Δ(*α*) = ∅, then *front*(Λ(*α*),Δ(*α*)) = *null* and, consequently, the sent value in the *backward* message will be *null*. Hence, by receiving a *null* value from a child, *α* can mark that link as a bridge. Therefore, each node can correctly decide whether a cross, child and parent link is a bridge or not.

### 5.2. Message Complexity

In the ABIDE algorithm, each node exactly sends one *forward* and one *backward* message. Hence, we can prove the following theorems about this algorithm. In the following theorems, *N* is the node count, *δ* is the maximum degree of the graph and *D* is the graph diameter.

#### Theorem 2

*The complexity of the sent message count of the ABIDE algorithm is O(N)*.

##### Proof

In this algorithm, all nodes send one *forward* message. Furthermore, all nodes, except the sink, send one *backward* message to their parents. Therefore, the total sent message count is 2*N*–1; therefore, the sent message complexity is *O*(*N*).

#### Theorem 3

*The complexity of the received message count of ABIDE algorithm is O(δN)*.

##### Proof

Due to the broadcast communication in wireless sensor networks, each node may multicast a message to its immediate neighbors. In the worst case, if we assume that each node has neighbor *δ*, then all nodes in the network, except the sink's neighbors, will receive *δ forward* and *δ backward* messages. Because the sink does not send a *backward* message, its neighbors will receive *δ* —1 *backward* messages. Therefore, the received message complexity is O(*δN*).

#### Theorem 4

*The complexity of message size in the ABIDE algorithm is O(D log_2_(N)) bits*.

##### Proof

At the forward stage of ABIDE, each node multicasts its ancestor list to its neighbors. The size of the ancestor list can be at most *D*-1. Because of this, the message size of ABIDE is O(*D*log_2_(*N*)) bits.

### 5.3. Time, Space and Computational Complexities

#### Theorem 5

*The time complexity of the ABIDE algorithm is O(D)*.

##### Proof

The ABIDE algorithm starts when the sink multicasts a *forward* message and finishes when the sink receives a *backward* message from all of its neighbors. *Forward* messages are flooded, until they are received by the leaf nodes in the BFS tree. Similarly, *backward* messages are convergecasted from leaf to sink. The maximum height of the BFS tree is *D;* hence, the time complexity of the ABIDE algorithm is O(*D*).

#### Theorem 6

*The space complexity of the ABIDE algorithm is O(D) per node*.

##### Proof

Each node must store its ancestor list. In the worst case, a leaf node must hold a list with *D*-1 ancestor information, because the maximum height of the BFS tree is *D*. Thus, the space complexity of the ABIDE algorithm is O(*D*) per node.

#### Theorem 7

*The computational complexity of the ABIDE algorithm is O(δD)*.

##### Proof

The computational complexity of most functions that are presented for algorithm implementation are O(1), except ⋏ and *front* operations. Each node calls ⋏ whenever it receives a *forward* message and calls the *front* function whenever it receives a *backward* message. Each node receives at most *δ forward* and *δ backward* messages; hence, the overall computational complexity of these two function is O(*δD*).

**Figure 6.**
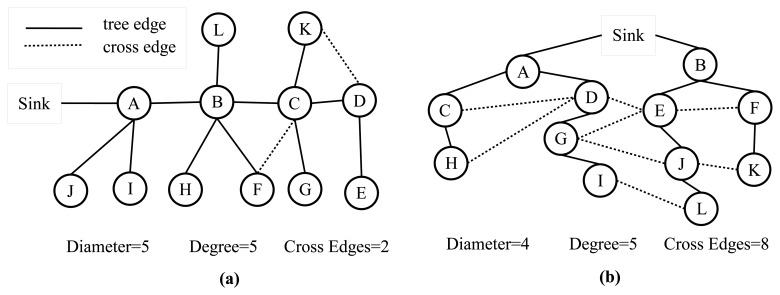
(**a**) Caterpillar-like graph; (**b**) random dense graph.

An analytical comparison between the TURAU algorithm, MILIC algorithm, E-MILIC algorithm and ABIDE algorithm is given in Table 1. MILIC, E-MILIC and ABIDE algorithms have O(*N*) sent message complexity and O(*δN*) received message complexity and are better than TURAU in terms of message transfer complexity. Although the message size of the TURAU is O(log_2_(*N*)), its received message complexity is O(*δE*). The message size of the ABIDE algorithm is O(*D*log_2_(*N*)), where *D* values are shown to be O(log_2_(*N*)) for scale-free networks [40]. In regards to this, we may state that the message size of the ABIDE algorithm is O(log^2^_2_(*N*)) for random networks. The message size of MILIC is O(ℰlog_2_(*N*)), where ℰ is the set of all cross edges. E-MILIC's message size is O(Ψlog_2_(*N*)), where Ψ is the set of cross edges that do not belong to a three-cycle. It is trivial to show that ℰ ∈ *E* and Ψ ∈ O(ℰ). The complexity of the transmitted byte counts are given in the fifth row of Table 1. For sparsely connected networks with Ψ ∈ O(*D*), then O(*δ*Ψ log_2_(*N*) ∈ O(*δ^2^E*log*_2_*(*N*)) and O(*δ*Ψ log_2_(*N*) ∈ O(*δD* log_2_(*N*)), which shows that E-MILIC is favorable. This case is true for such sparse caterpillar-like graphs, whose subgraph, formed by deleting cross edges, is a caterpillar tree, as shown in Figure 6a. In a caterpillar tree, each vertex is on the central path or its distance to the central path is one. For densely connected sensor networks with log_2_(*N*) ∈ *D* ∈ O(Ψ), then O(*δD* log_2_(*N*)) ∈ O(*δ^2^E*log_2_(*N*)) and O(*δD* log_2_(*N*)) ∈ O(*δ*Ψlog_2_(*N*)), which shows that ABIDE is favorable. This case is true for random and dense graphs, as shown in Figure 6b. The time complexity of the ABIDE algorithm is O(*D*), and same as MILIC and E-MILIC, the space and the computational complexities of the ABIDE algorithm depend on *D*. On the other hand, TURAU has O(*N*) time complexity; MILIC and E-MILIC algorithms have O(*E*) space and computational complexities. In the next section, we will give testbed experiments and extensive simulation results to show the practical importance of the proposed algorithms.

## 6. Performance Evaluations

In this section, we show extensive performance evaluations of the algorithms by giving testbed experiments and simulations.

### 6.1. Experiments

To evaluate the performance of our algorithms, we firstly conduct experiments on our testbed. The proposed algorithms, E-MILIC and ABIDE, are implemented with their counterparts: MILIC and TURAU algorithms. The algorithms are implemented in nesC language for TinyOS platform [41] running on Crossbow-Memsic IRISmotes. The IRIS is a 2.4 GHz mote with an IEEE802.15.4 [25] compliant transceiver. It has 128 kB programmable flash memory, 8 kB RAM, 250 kpbs nominal data rate and 3 dBm transmission power. A TDMA-based MAC protocol is implemented. The features of the motes are summarized in Table 2. The indoor range of an IRIS mote is up to 50 m, but it can be adjusted by reducing transmission power and receiving sensitivity. Twenty motes are used in the experiment testbed. The algorithms have been evaluated in sparse and dense topologies. In a sparse topology, each node has three neighbors, and in a dense topology, each node has seven neighbors, on average. Additionally, the algorithms have been evaluated in caterpillar-like topologies.

**Figure 7.**
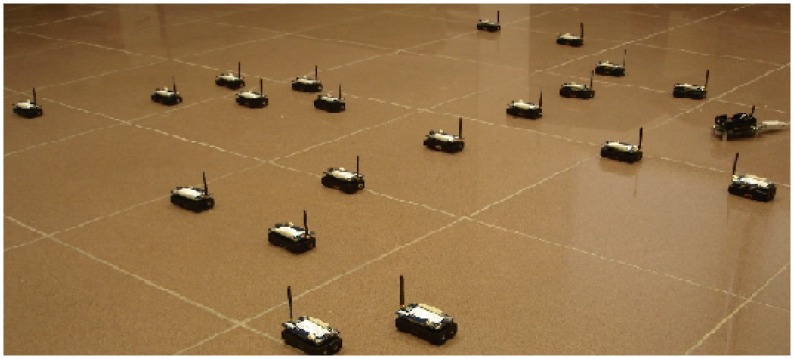
The deployment of the sparse topology.

**Figure 8.**
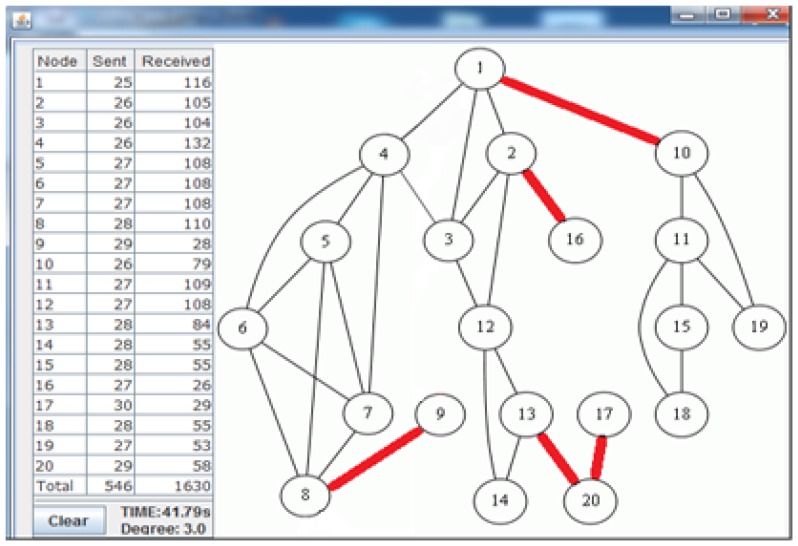
Snapshot of the monitoring program for the sparse topology.

**Figure 9.**
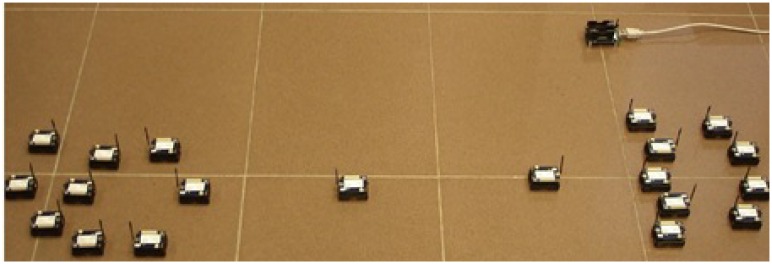
The deployment of the dense topology.

**Figure 10.**
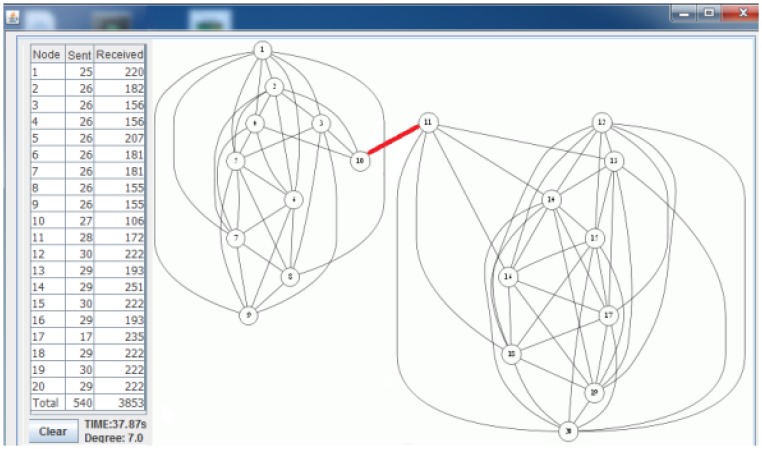
Snapshot of the monitoring program for the dense topology.

Figure 7 shows the deployed sparse topology in our laboratory. A monitoring program is written in Java language, which shows connections between nodes with the sent byte counts, received byte counts, wall clock times, average node degrees and the list of message transfers. The snapshot of the monitoring program for the sparse topology is given in Figure 8. The bridges are shown with bold and red edges. The dense deployment is given in Figure 9, where its graph is given in Figure 10.

Table 3 shows the performance of the algorithms in the sparse deployment. The experimental results show that the proposed improvements in E-MILIC over MILIC lead to better performance in sent and received bytes. The sent byte count of E-MILIC is 17% lower than MILIC, and the received byte count of E-MILIC is 18% lower than TURAU. TURAU has the worst performance in terms of all metrics, since it uses unicast messages. ABIDE has the best performance in terms of all metrics. The sent byte count of ABIDE is 6% lower than E-MILIC, 22% lower than MILIC and 45% lower than TURAU. The received byte count of ABIDE is 8% lower than E-MILIC, 25% lower than MILIC and 40% lower than TURAU.

Table 4 shows the performance of the algorithms when the topology is dense. Obviously, in dense topologies, the heights of the BFS tree are less than the heights of the BFS trees in sparse topologies. Hence, the differences between ABIDE and other algorithms in terms of sent and received bytes are more than those in sparse graphs. The received and sent byte counts of ABIDE are almost half of the MILIC and a quarter of the TURAU algorithms. Except the TURAU algorithm, which uses unicast messages, all other algorithms use radio multicast messages and send only *forward* messages and *backward* messages; hence, their wall clock times are almost equal.

**Figure 11.**
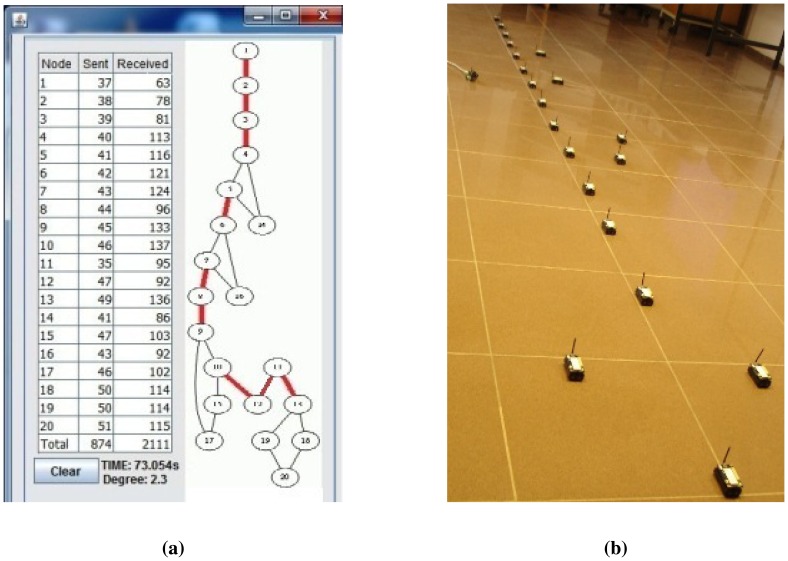
Snapshot of the monitoring program and deployment for the caterpillar topology. (**a**) The deployment; (**b**) snapshot of the monitoring program.

Lastly, we measured the performance of the algorithms for a caterpillar-like topology. In Figure 11a, the caterpillar-like deployment is given, and its graph is given in Figure 11b. The related measurements are given in Table 5. The diameter of this topology is high and its cross edge count is low compared to the previous dense and sparse random topologies. Therefore, E-MILIC performs the best in terms of sent byte counts, received byte counts and energy consumption. The received byte count of E-MILIC is 8% lower than TURAU, 10% lower than ABIDE and 34% lower than MILIC. The wall clock times of E-MILIC, MILIC and TURAU are similar, since they are BFS-based; on the other side, the performance of TURAU is far worse than the other algorithms, since it is DFS-based.

ABIDE performs best for random graphs, and E-MILIC performs best for caterpillar-like graphs. Although these experiments give ideas about the performance of algorithms and their efficiency, the size of the network is limited with 20 nodes. Besides, TinyOS does not provide an interface to measure energy consumption. In order to evaluate algorithms for large-scale networks, we use a simulator in the following section.

### 6.2. Simulations

We implemented ABIDE, E-MILIC, MILIC and TURAU algorithms in a TOSSIMsimulator [41], which is a TinyOS simulator for WSNs. The programs, written in nesC language, can be directly simulated in TOSSIM with minor modifications. We have simulated all algorithms for various network sizes, from 50 to 250 nodes. The transmission range of sensor nodes is 50 m. We measured received byte counts, sent byte counts, energy consumption and wall clock times. Each measurement is the average of 10 repeated simulations, where standard deviations are very small compared to the means. Random graphs and caterpillar-like graphs are used in the simulations. We implemented a TDMA-based MAC protocol, and we used an IEEE 802.15.4 physical layer for the lower layers. The simulation parameters are summarized in Table 6. Random graphs are generated by randomly placing the sink node and ordinary nodes in the area for each simulations in Section 6.2.1, Section 6.2.2, Section 6.2.3 and Section 6.2.4. In these simulations, the degrees are varied between three, five and seven. Caterpillar-like graphs are generated in Section 6.2.5.

#### 6.2.1. Sent Byte Counts in Random Graphs

**Figure 12.**
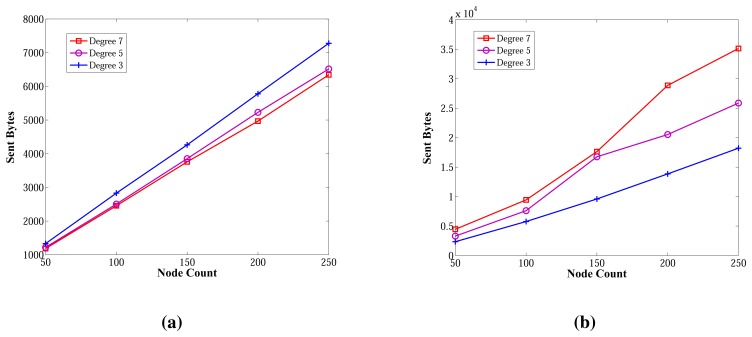
Sent bytes of ABIDE and E-MILIC algorithms against node count and degree. (**a**) Sent bytes of ABIDE; (**b**) sent bytes of E-MILIC.

The sent byte counts of ABIDE and the sent byte counts of E-MILIC algorithms against various node counts and degrees are shown in Figure 12a,b, respectively. When node counts and degrees are increased linearly, the sent byte count values of the ABIDE and E-MILIC algorithms show a linear increase. This shows that the algorithms are stable and scalable in terms of sent byte counts.

**Figure 13.**
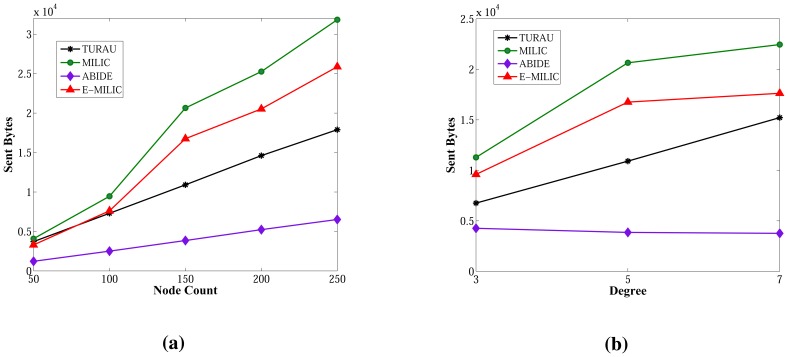
Comparisons between algorithms in terms of sent bytes. (**a**) Sent bytes against node count; (**b**) sent bytes against degree.

Comparisons between the implemented algorithms in terms of sent byte counts are given in Figure 13a,b. E-MILIC performs better than MILIC for all simulation setups. As shown in Figure 13a, in small networks, where the node count is less than 100, the total sent byte counts of E-MILIC are less than TURAU. However, for networks with more than 100 nodes, TURAU performs better. The reason is that E-MILIC transmits cross edge information; on the other side, the message size of TURAU is constant and O(log_2_(*N*)) bits. ABIDE performs best among the other algorithms, where the sent byte count of ABIDE is approximately up to three-times better than TURAU, four-times better than E-MILIC and five-times better than the MILIC algorithm, as shown in Figure 13a. Moreover, when the degree is increased, the sent byte counts of the ABIDE algorithm decrease, whereas the sent byte counts of other algorithms increase, as shown in Figure 13b.

#### 6.2.2. Received Byte Counts in Random Graphs

**Figure 14.**
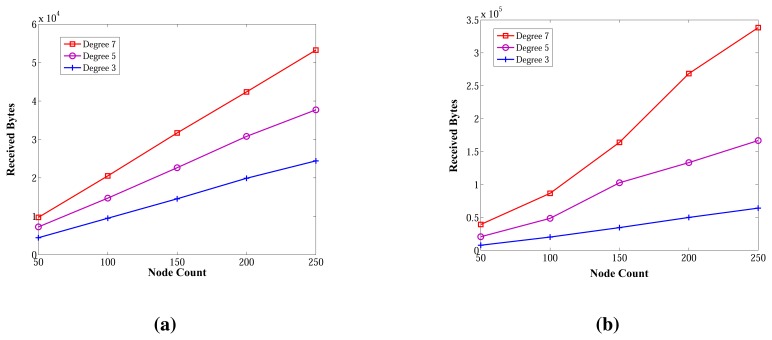
Received bytes of ABIDE and E-MILIC algorithms against node count and degree. (**a**) Received bytes of ABIDE; (**b**) received bytes of E-MILIC.

Figure 14 shows the received byte counts of the ABIDE and E-MILIC algorithms against node count and degree. As shown in Figure 14a, when node count is increased, the received byte counts of the ABIDE algorithm increase linearly for all degree values. This shows us that the ABIDE algorithm is stable and scalable in terms of received byte counts. On the other side, the received byte counts of the E-MILIC algorithm do not show a linear increase when the degree is seven, as shown in Figure 14b. This situation shows that the proposed rules for the E-MILIC algorithm do not perform well for dense topologies.

**Figure 15.**
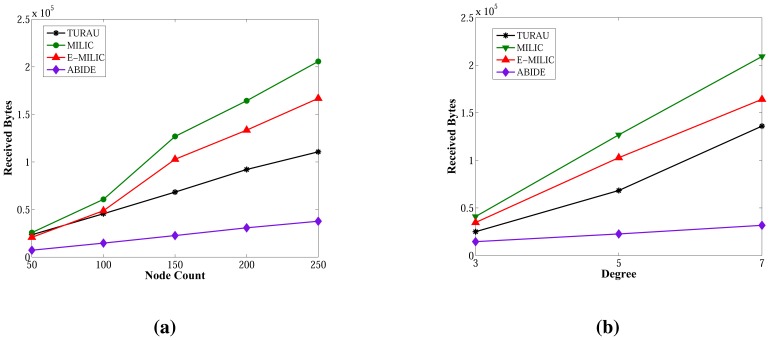
Comparisons between algorithms in terms of received bytes. (**a**) Received bytes against node count; (**b**) received bytes against degree.

Comparisons between algorithms in terms of received bytes are given in Figure 15. The comparisons show that ABIDE has the best performance. E-MILIC outperforms MILIC, but TURAU performs better than E-MILIC when the network size is greater than 100 nodes, as shown in Figure 15a,b. The received byte count of ABIDE is approximately 57% less than TURAU, 72% less than E-MILIC and 77% less than MILIC. This is a significant improvement for decreasing energy consumption caused by the receiving operations. In the following section, we will give the energy consumption measurements.

#### 6.2.3. Energy Consumption in Random Graphs

**Figure 16.**
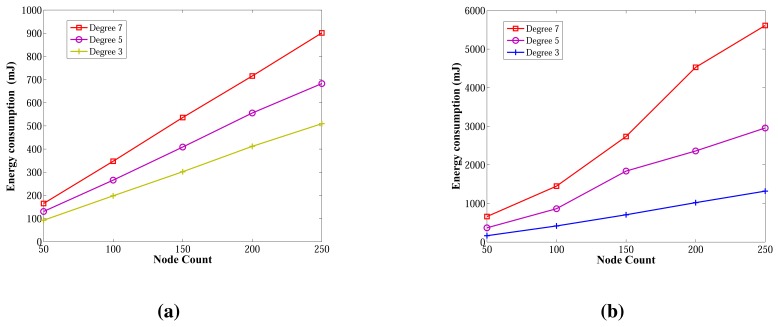
Energy consumption of the ABIDE and E-MILIC algorithms against node count and degree. (**a**) Energy consumption of ABIDE; (**b**) energy consumption of E-MILIC.

Energy consumption measurements for the ABIDE and E-MILIC algorithms for networks with various sizes and degrees are shown in Figure 16a,b, respectively. The energy consumption of ABIDE shows a linear growth against node count and degree. Although the energy consumption of E-MILIC grows linearly for sparse networks, the slope of the line increases when the degree increases.

**Figure 17.**
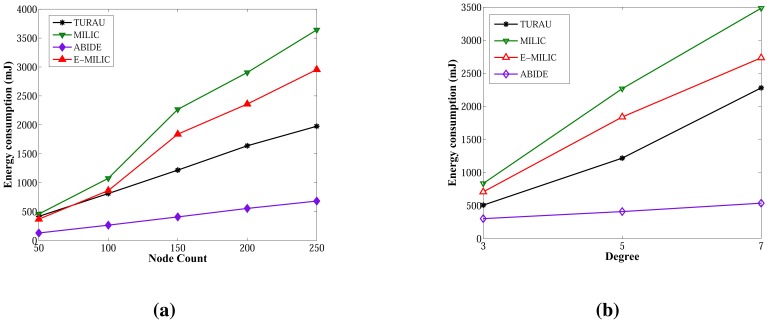
Comparisons between algorithms in terms of energy consumption. (**a**) Energy consumption against node count; (**b**) energy consumption against degree.

Comparisons between the ABIDE, E-MILIC, MILIC and TURAU algorithms in terms of energy consumption are given in Figure 17. E-MILIC approximately consumes 17% less energy than MILIC, on average. TURAU performs better than E-MILIC when the node count is greater than 100, as explained in the previous sections. ABIDE shows the best performance, and its energy consumption is approximately three-times better than TURAU, 4.5-times better than E-MILIC and 5.5-times better than MILIC, on average, as shown in Figure 17a. Besides, ABIDE is stable against degree values and shows a linear increase with a very small slope, as shown in Figure 17b. This is a significant contribution to the energy-efficient bridge detection problem in WSNs when the topology is random.

#### 6.2.4. Wall Clock Times in Random Graphs

**Figure 18.**
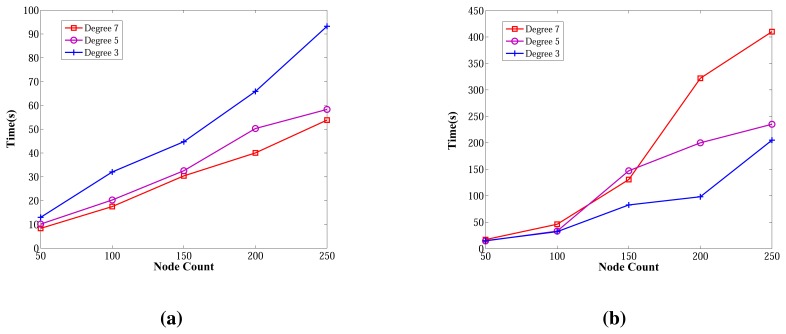
Wall clock times of ABIDE and E-MILIC algorithms against node count and degree. (**a**) Wall clock times of ABIDE; (**b**) wall clock times of E-MILIC.

The wall clock times of ABIDE and E-MILIC algorithms against various node counts and degrees are shown in Figure 18. The ABIDE algorithm is stable and scalable against node count and degree, since it is fully integrated into the BFS, and the transmitted packet sizes are O(*D*log_2_(*N*)) bits. E-MILIC performs well for the networks when the degree equals three, since it is fully integrated into the BFS, like the ABIDE algorithm, but the wall clock time significantly increases with the degree, since the transmitted packet sizes are O(Ψlog_2_(*N*)) bits.

**Figure 19.**
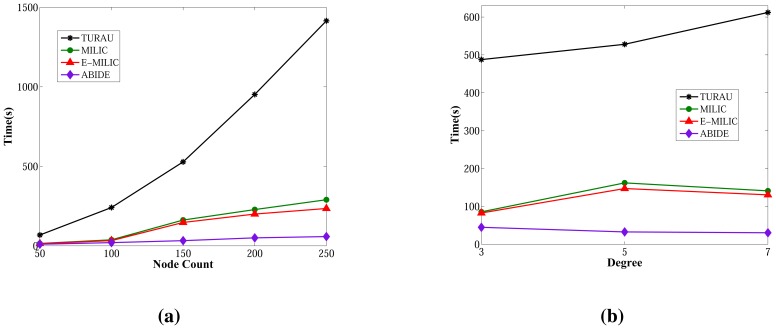
Comparisons between algorithms in terms of wall clock times. (**a**) Wall clock times against node count; (**b**) wall clock times against degree.

Figure 19 gives the comparisons between algorithms in terms of wall clock times. Unlike the previous measurements, TURAU is the worst algorithm. Its performance is significantly worse than the other algorithms. In the worst case, it runs approximately 28-times slower than the ABIDE algorithm. The reason is that TURAU uses unicast messages between nodes in each step, which leads to it consuming a huge running time to complete its execution in large-scale networks. E-MILIC is faster than MILIC in all cases where the wall clock time differences between them are very small. ABIDE has the best performance, which can be seen in Figure 19a,b. The algorithmic ideas proposed in the ABIDE algorithm suit the randomly deployed WSN environment and provides significant time savings.

#### 6.2.5. Measurements in Caterpillar-Like Graphs

**Figure 20.**
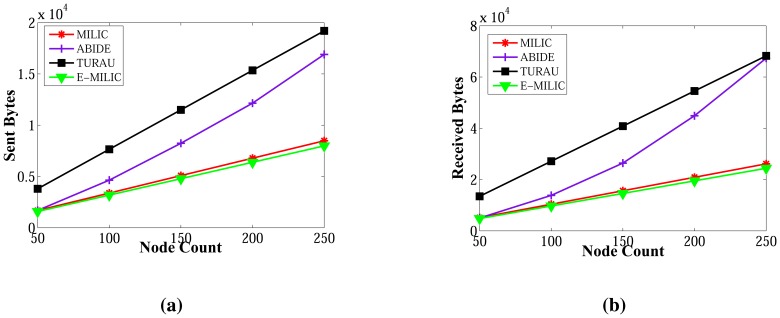
Comparisons between algorithms in terms of sent byte counts and received byte counts for caterpillar-like graphs. (**a**) Sent byte counts against node count; (**b**) received byte counts against node count.

Lastly, we measured the sent byte counts, received byte counts, energy consumption and wall clock times on caterpillar-like graphs. The sent byte counts and the received byte counts of the algorithms against various node counts are shown in Figure 20a,b, respectively. The performance order of the algorithms for both figures is: E-MILIC, MILIC, ABIDE and TURAU. TURAU has the worst performance, since it uses unicast communication on caterpillar-like topology. Since the diameter of the graphs are high and cross edge count is low, ABIDE has the second worst performance. E-MILIC performs the best, where its received byte count is approximately 6% less than MILIC, 44% less than ABIDE and 64% less than TURAU, on average. The reason for this fact is that the proposed rules in E-MILIC provide transmission of small messages when cross edge count is low.

**Figure 21.**
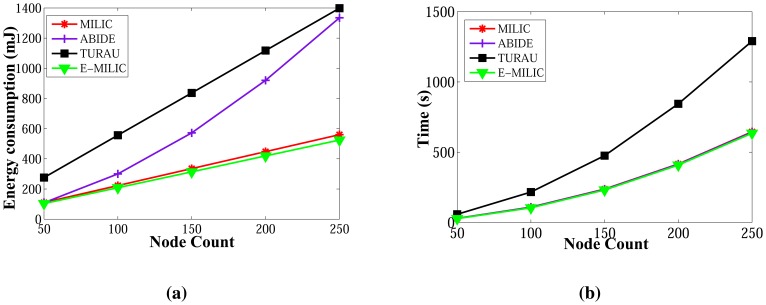
Comparisons between algorithms in terms of energy consumption and wall clock times for caterpillar-like graphs. (**a**) Energy consumption against node count; (**b**) wall clock times against node count.

Comparisons between the algorithms in terms of energy consumption and wall clock times are given in Figure 21a,b, respectively. E-MILIC has the best performance in energy consumption, since its sent byte count and received byte count are better than those of its counterparts. E-MILIC consumes 2.09 mJ per node, MILIC consumes 2.24 mJ per node, ABIDE consumes 3.81 mJ per node and TURAU consumes 5.78 mJ per node. Wall clock times of E-MILIC are best among other algorithms; MILIC and ABIDE have similar performance, since they are BFS-based algorithms. TURAU is a DFS-based algorithm, and it has the worst performance.

In this section, we showed from the measurements taken that our analytical results given in Section 5 conform with the results taken from our testbed experiments and simulation results; E-MILIC performs better than the former MILIC algorithm. ABIDE has the best performance for random graphs and E-MILIC has the best performance for caterpillar-like graphs in terms of all metrics.

## 7. Conclusions

In this paper, we propose two BFS-based and single-phase bridge detection algorithms for WSNs located on challenged environments. Our first proposed algorithm, E-MILIC, is the extended version of the MILIC algorithm. In this algorithm, two rules are added to the MILIC algorithm to reduce the length of the messages. The sent message complexity of E-MILIC is Θ(*N*), the received message complexity is O(*δN*), the message size is O(Ψlog_2_(*N*)), the transmitted byte count is O(*δ*Ψlog_2_(*N*)), the time complexity is O(*D*) and the space and computational complexities are O(*E*). Our second proposed algorithm, ABIDE, uses ancestral knowledge to detect bridges. This idea is novel for bridge detection in sensor networks. Theoretically, the ABIDE algorithm has Θ(*N*) sent message complexity, O(*δN*) received and overheard message complexity, O(*δD*log_2_(*N*)) transmitted byte count, where each message is O(*D*log_2_(*N*)) bits, O(*D*) time and space complexities and O(*δD*) computational complexities.

We gave the analysis for the proof of correctness and the message, time, space and computational complexities of the algorithms. Both algorithms are implemented in an experiment testbed and a simulation environment with their counterparts. Measurements conform with the theoretical analysis that the proposed algorithms outperform their counterparts, both practically and theoretically. By applying the proposed rules, E-MILIC has the best performance in terms of the received byte count, sent byte count, energy consumption and wall clock times, among other algorithms for caterpillar-like graphs. For random graphs, ABIDE has the best performance in terms of all metrics, since it uses smaller messages to detect bridges. The time savings of ABIDE are up to 28-times and energy savings up to 5.5-times for random graphs. Our proposed algorithms are significant contributions to the resource-efficient bridge detection problem in sensor networks operating in harsh environments.

## Figures and Tables

**Table 1.** Analytical comparison of algorithms.

**Complexity/Algorithm**	**TURAU**	**MILIC**	**E-MILIC**	**ABIDE**
Sent Messages	Θ(*E*)	Θ(*N*)	Θ(*N*)	Θ(*N*)
Received Messages	O(*δE*)	Θ(*δN*)	Θ(*δN*)	Θ(*δN*)
Message Size	O(log_2_(*N*))	O(ℰ log_2_(*N*))	O(Ψ log_2_(*N*))	O(*D* log_2_(*N*))
Transmitted Byte Count	O(*δ*^2^*E*log_2_(*N*))	O(*δ*ℰ log_2_(*N*))	O(*δ*Ψ log_2_(*N*))	O(*δD* log_2_(*N*))
Time	O(*N*)	O(*D*)	O(*D*)	O(*D*)
Space (per node)	O(*E*)	O(*E*)	O(*E*)	O(*D*)
Computational (per node)	O(δ)	O(*E*)	O(*E*)	O(*δD*)

**Table 2.** Experiment Testbed Parameters.

**Mote**	**IRIS**
Platform	TinyOS
Transceiver	2.4 GHz, IEEE 802.15.4 compliant
Transmission Rate	250 kbps
Transmission Power	3 dbm
Memory	128 kB flash, 8 kB RAM
Node Count	20
Node Degree	3 and 7

**Table 3.** Performance of algorithms in sparse topology.

**Algorithm/Performance**	**Sent Bytes**	**Received Bytes**	**Wall Clock Times (s)**
MILIC	701	2,189	41.797
E-MILIC	581	1,778	41.798
TURAU	990	2,739	256.63
ABIDE	546	1,630	41.793

**Table 4.** Performance of algorithms in dense topology.

**Algorithm/Performance**	**Sent Bytes**	**Received Bytes**	**Wall Clock Times (s)**
MILIC	816	6,209	38.361
E-MILIC	579	4,250	37.967
TURAU	1859	11,986	514.418
ABIDE	540	3,853	37.878

**Table 5.** Performance of algorithms in caterpillar-like topology.

**Algorithm/performance**	**Sent Bytes**	**Received Bytes**	**Wall Clock Times (s)**
MILIC	1,059	2,539	73.32
E-MILIC	844	1,891	73.04
TURAU	936	2,057	178.50
ABIDE	874	2,111	73.05

**Table 6.** Simulation parameters.

Topology	Random and Caterpillar-like
Sink Position	Randomly Placed in the Area
Sensor Node Count	50–250
MAC	TDMA
Node Degrees	3, 5 and 7
